# Supplementary material for: Two-Dimensional Selenium Nanosheet-Based Sponges with Superior Hydrophobicity and Excellent Photothermal Performance
Source: Nanomaterials (Basel). 2022 Oct 26;12(21):3756. doi: 10.3390/nano12213756 (PMC9657928; doi:10.3390/nano12213756)
Supplement: Supplementary file 1 [file nanomaterials-12-03756-s001.zip › nanomaterials-1960949-supplementary.pdf]

Two-Dimensional Selenium Nanosheet-Based Sponges with Superior Hydrophobicity and Excellent Photothermal Performance

Hongyan Chen [1], Mengke Wang [2,*] and Weichun Huang [2,*]

[1] Engineering Training Center, Nantong University, Nantong 226019, China
[2] School of Chemistry and Chemical Engineering, Nantong University, Nantong 226019, China
* Correspondence: mengkewang@ntu.edu.cn (M.W.); huangweichun@ntu.edu.cn (W.H.)

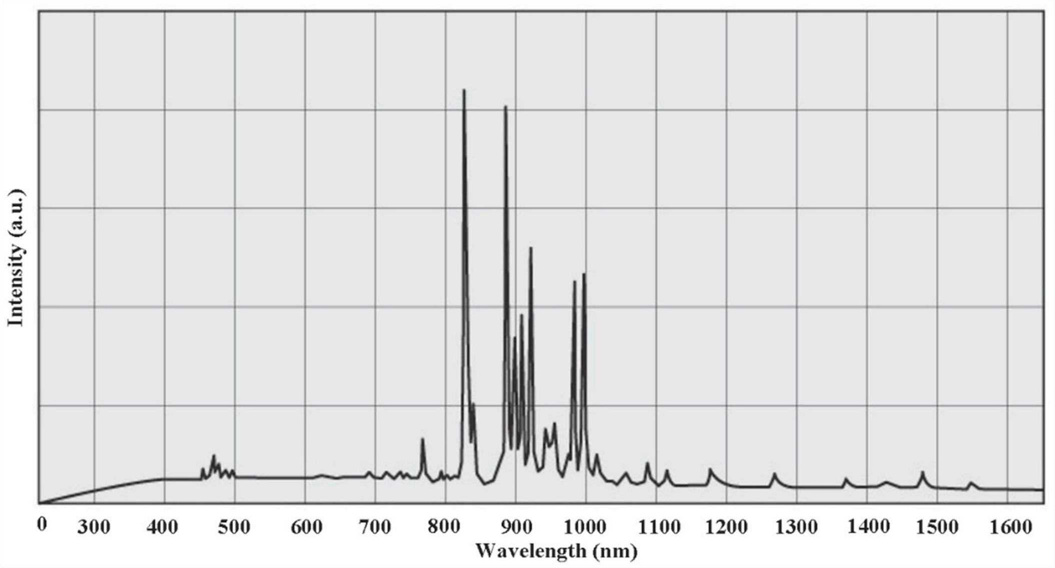

Figure S1. Wavelength of the Xe short arc lamp solar simulator.

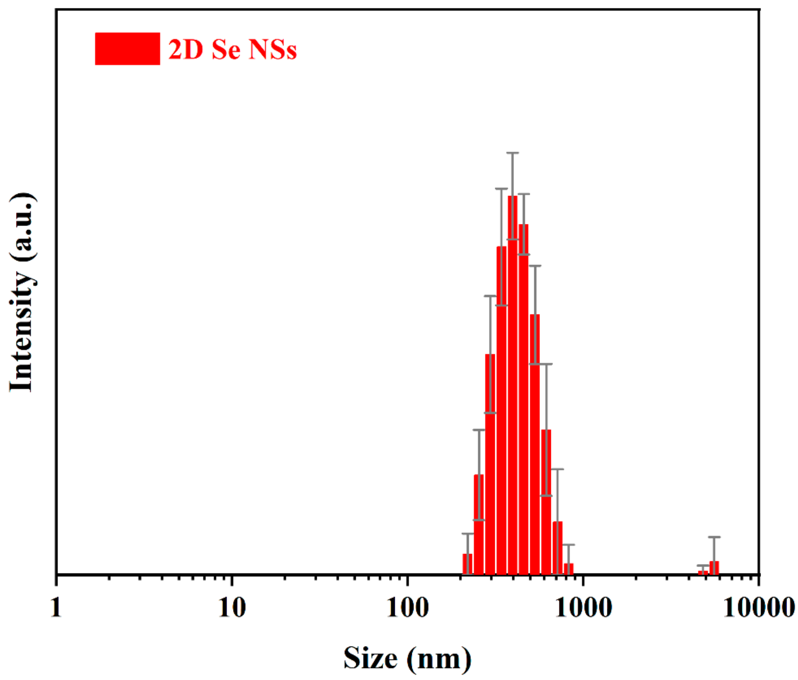

Figure S2. Size distribution of the Se NSs.

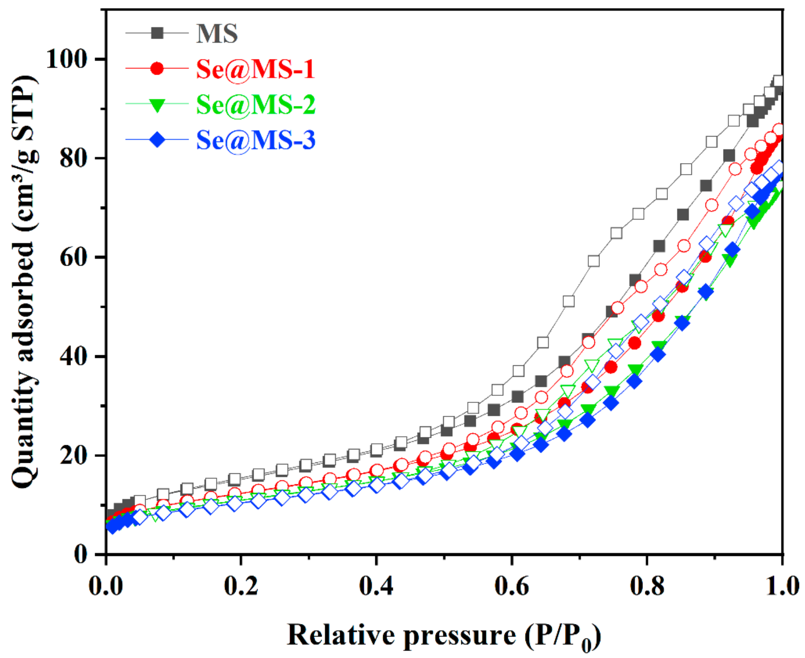

Figure S3. N_2 Isotherm linear adsorption and desorption curves for the pristine MS, Se@MS-1, Se@MS-2, and Se@MS@-3.

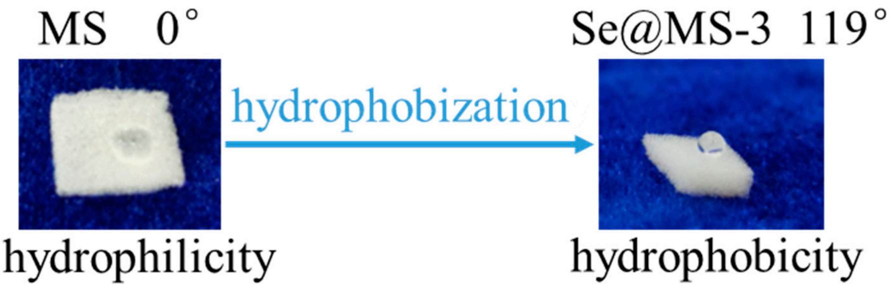

Figure S4. Wettability of a water droplet on the pristine MS and the as-fabricated Se@MS-3.

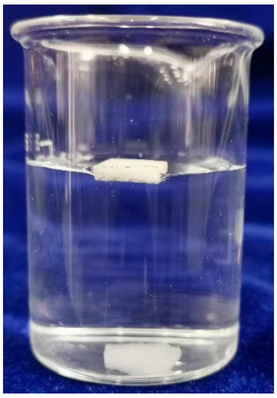

Figure S5. Optical image of the pristine MS and Se@MS interacting with water.

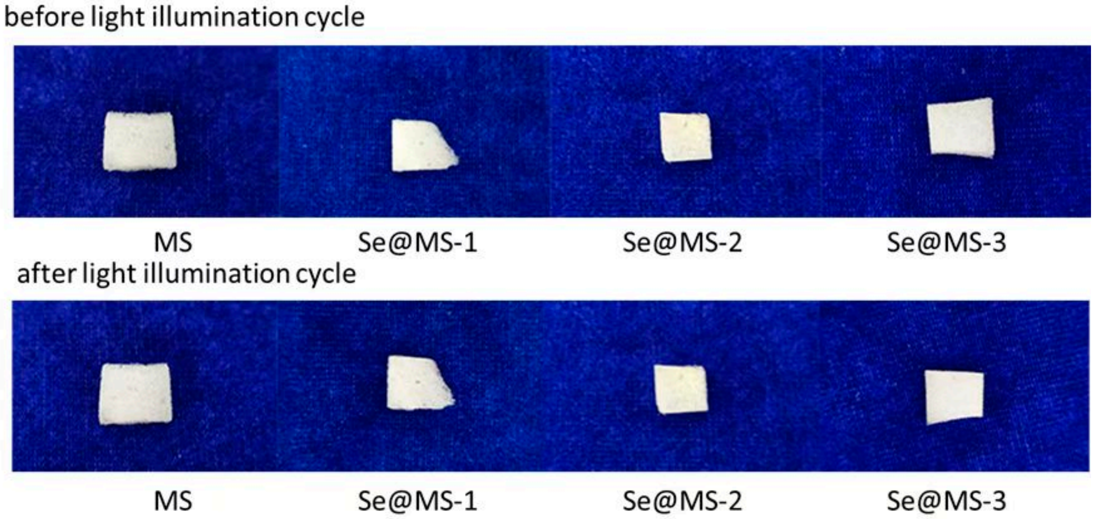

Figure S6. Photos of the as-fabricated Se@MS-2 before and after light illumination cycle with a power density of 1.0 W cm^{-2}.
